# A Fisetin Delivery System for Neuroprotection: A Co-Amorphous Dispersion Prepared in Supercritical Carbon Dioxide

**DOI:** 10.3390/antiox13010024

**Published:** 2023-12-21

**Authors:** Szymon Sip, Natalia Rosiak, Anna Sip, Marcin Żarowski, Katarzyna Hojan, Judyta Cielecka-Piontek

**Affiliations:** 1 Department of Pharmacognosy and Biomaterials, Poznan University of Medical Sciences, Rokietnicka 3, 60-806 Poznan, Poland; szymonsip@ump.edu.pl (S.S.); nrosiak@ump.edu.pl (N.R.); 2 Department of Biotechnology and Food Microbiology, Poznan University of Life Sciences, Wojska Polskiego 48, 60-627 Poznan, Poland; anna.sip@up.poznan.pl; 3 Department of Developmental Neurology, Poznan University of Medical Sciences, Przybyszewski 49, 60-355 Poznan, Poland; zarowski@ump.edu.pl; 4 Department of Occupational Therapy, Poznan University of Medical Sciences, 60-781 Poznan, Poland; khojan@ump.edu.pl; 5 Department of Rehabilitation, Greater Poland Cancer Centre, 61-866 Poznan, Poland

**Keywords:** fisetin, amorphous, supercritical CO_2_, solubility, microbiome, neuroprotection

## Abstract

Fisetin (FIS), a senolytic flavonoid, mitigates age-related neuroprotective changes. An amorphous FIS dispersion with a co-carrier was prepared using supercritical fluid extraction with carbon dioxide (scCO_2_). Characterisation, including powder X-ray diffraction and Fourier-transform infrared spectroscopy, confirmed amorphization and assessed intermolecular interactions. The amorphous FIS dispersion exhibited enhanced solubility, dissolution profiles, and bioavailability compared to the crystalline form. In vitro, the amorphous FIS dispersion demonstrated antioxidant activity (the ABTS, CUPRAC, DDPH, FRAP assays) and neuroprotective effects by inhibiting acetylcholinesterase and butyrylcholinesterase. FIS modulated gut microbiota, reducing potentially pathogenic gram-negative bacteria without affecting probiotic microflora. These improvements in solubility, antioxidant and neuroprotective activities, and gut microbiome modulation suggest the potential for optimising FIS delivery systems to leverage its health-promoting properties while addressing oral functionality limitations.

## 1. Introduction

**Figure 1:**
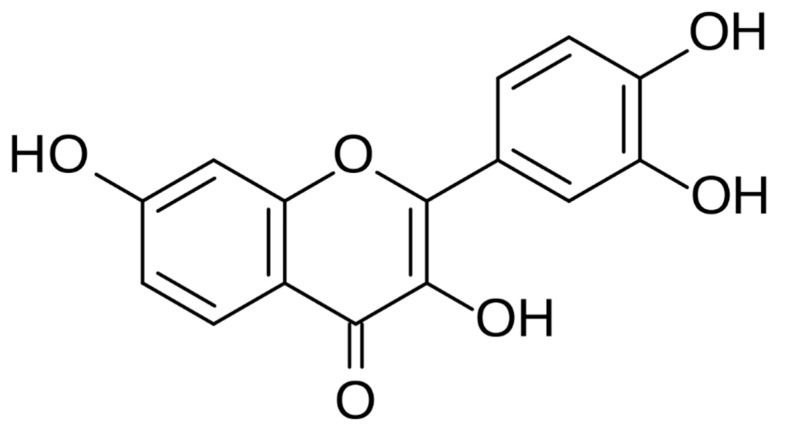
The structural formula of fisetin.

Fisetin (FIS) is a naturally occurring senolytic flavonoid compound that belongs to the class of polyphenols with senolytic properties (Figure 1). It is commonly found in various fruits and vegetables, such as strawberries, apples, grapes, and onions [1,2]. Senolytics are compounds known for targeting and eliminating senescent cells, aged or damaged cells that accumulate in tissues over time and are associated with ageing and various age-related diseases [3,4]. It has a remarkable potential to selectively induce apoptosis (cell death) in senescent cells while leaving healthy cells unaffected [5]. Research has shown that FIS is considered one of the most potent senolytic compounds, exhibiting strong anti-senescence effects in preclinical studies across different tissues and animal models [6,7,8]. Its unique ability to promote the clearance of senescent cells has sparked interest in exploring FIS’s therapeutic applications for extending health span and potentially addressing age-related ailments [9].

FIS’s senolytic activity within the central nervous system (CNS) holds significant promise for addressing various age-related neurodegenerative conditions, including Alzheimer’s and Parkinson’s [10,11,12]. Senolytics are compounds that selectively induce apoptosis, or programmed cell death, in senescent cells, which have stopped dividing and are often associated with ageing-related pathologies. FIS’s unique senolytic action in the CNS is achieved by targeting specific anti-apoptotic proteins, such as Bcl-2 and Bcl-xl. By inhibiting these proteins, FIS sensitises senescent cells to programmed cell death, effectively eliminating them. This property offers a novel approach to reducing the burden of cellular dysfunction in the ageing brain, potentially slowing the progression of age-related cognitive decline and neurodegeneration. The potential applications of FIS’s senolytic effects within the CNS are diverse. They may be part of innovative interventions to maintain neurological health and improve the quality of life for individuals with age-related neurological conditions. FIS’s role in promoting programmed cell death in senescent cells is a promising area of research, suggesting it could play a significant role in future age-related therapeutic strategies for the CNS [13,14,15].

Furthermore, the research into FIS’s impact on CNS senescence has the potential to provide exciting opportunities for the development of treatments that enhance brain function and mitigate the effects of ageing on the nervous system. In summary, FIS’s senolytic activity in the CNS represents a hopeful avenue for addressing age-related neurological challenges and improving the overall well-being of affected individuals [16,17].

In addition to its well-documented neuroprotective effects, FIS has recently garnered attention for its potential utility in stroke management [18]. Stroke, a leading cause of disability and mortality worldwide, necessitates innovative therapeutic strategies [18]. With its antioxidative and anti-inflammatory properties, FIS presents a compelling candidate for stroke intervention. Recent preclinical studies have demonstrated FIS’s ability to mitigate the consequences of cerebral ischemia, offering neuroprotection by attenuating oxidative stress, reducing inflammation, and preserving neuronal integrity [19,20]. These findings suggest that FIS could play a pivotal role in post-stroke recovery by safeguarding against ischemic damage. Considering the limited therapeutic options for stroke patients, exploring FIS’s neuroprotective potential in the context of cerebrovascular events could open new avenues for effective interventions.

The discussion of advanced glycation end products (AGEs), their role in neurodegenerative diseases, and the potential protective effects of polyphenols, such as fisetin, presents a fascinating avenue for exploring preventive strategies. The formation of AGEs, resulting from non-enzymatic reactions during the Maillard process, is intricately linked to carbonyl stress and implicated in the pathogenesis of neurodegenerative disorders [21]. The interaction between AGEs and their receptors (RAGE) triggers oxidative stress, inflammation, and activation of signalling pathways associated with these diseases. Polyphenols, characterised by multiple hydroxyl groups, emerge as promising agents in combating AGEs-related neurodegeneration. Their ability to scavenge reactive oxygen species (ROS) and trap α-dicarbonyl species interrupts the formation of AGEs, offering a multifaceted approach to prevention. Specifically, polyphenols like fisetin demonstrate the capacity to inhibit AGEs production, disrupt RAGE–ligand interactions, and modulate gut microbiota. The connection between neurodegenerative diseases and gut microbiota imbalance is a pivotal aspect. Dietary polyphenols, including fisetin, showcase the potential to regulate the microbiota-gut-brain axis. By enhancing the abundance and diversity of gut microbiota, polyphenols contribute to intestinal inflammation alleviation, thereby delaying or preventing the progression of neurodegenerative diseases [22]. With its documented neuroprotective and antioxidant properties, supplementation with fisetin emerges as a strategic and promising intervention. Fisetin’s multifunctional effects on AGEs inhibition, its role in disrupting RAGE–ligand interactions, and its impact on gut microbiota collectively position it as a valuable candidate for preventing neurodegenerative diseases [23]. This aligns with the broader strategy of supplementing dietary polyphenols to modulate the AGEs–RAGE and microbiota–gut–brain axes, presenting a holistic and forward-thinking approach to neuroprotection.

Despite the promising therapeutic potential of FIS, its clinical application has been hindered by its low solubility in water [24,25]. This limited solubility reduces bioavailability, making achieving therapeutic concentrations of FIS in the body challenging. Consequently, researchers have explored various strategies to enhance the solubility of FIS, aiming to optimise its delivery and efficacy. In this review, the authors collected the most popular techniques for improving the solubility of FIS [26]. One approach involves the utilisation of nanoparticles, which can encapsulate FIS and improve its solubility while also providing controlled release properties [27,28]. Liposomes, another strategy, involve encapsulating FIS within lipid-based vesicles, enhancing its stability and solubility. These drug delivery systems facilitate the efficient transport and absorption of FIS in the body, potentially leading to improved therapeutic outcomes [29,30]. At the same time, amorphization is a process that converts a crystalline compound into an amorphous form, which can significantly improve its solubility and bioavailability [31]. This is because the amorphous form lacks the ordered crystalline structure, leading to higher energy and increased molecular mobility, making it easier to dissolve in solvents and facilitating absorption by the body [32,33,34]. However, traditional methods of amorphization, such as spray-drying and ball milling, have limitations, including the use of toxic solvents, the generation of waste, and the potential degradation of the compound. A new approach to amorphization is using co-precipitation with the functional support of carriers in supercritical carbon dioxide. This technique has many advantages regarding its ecological nature, the possibility of scaling the process, and low costs. Using CO_2_ in a supercritical state is a green and innovative method that has been demonstrated to be effective in amorphized compounds while overcoming the limitations of traditional methods [35,36].

Therefore, this work aimed to obtain an amorphous dispersion of fistin using co-precipitation with a functional carrier in flowing supercritical carbon dioxide. For the obtained amorphous dispersions of FIS, their functionality was assessed for its potential antioxidant, neuroprotective, and intestinal microbiota-modifying effects.

## 2. Materials and Methods

### 2.1. Fisetin Delivery System Preparation

FIS systems with copovidone (CPV) were prepared by preparing physical mixtures and pounding each mixture for 5 min in an agate mortar. Then, 5.0 g of the samples was transferred to a steel vessel. The vessel was then connected to an SFT-120 apparatus (Supercritical Fluid Technologies, Inc., Newark, DE, USA). The process parameters were determined as part of the preliminary tests; the optimal conditions were the pressure of 6000 psi and the temperature of the vessel at 80 °C. The process was carried out for 3 h. After the process was completed, CO_2_ flowed through the system. The sample was removed from the vessel and ground using a Tube Mill 100 control (IKA, Warsaw, Poland) for 1 min at 8000 rpm to homogenise it. The physical mixtures for each sample treated with scCO_2_ were also tested as controls. As a result of the work carried out, four systems were subjected to the process, and four physical mixtures were obtained. The tested systems differed in the content of FIS:

▪ SC 1 (10% fisetin)—sample treated with scCO_2_;
▪ PM 1 (10% fisetin)—physical mixture;
▪ SC 2 (15% fisetin)—sample treated with scCO_2_;
▪ PM 2 (15% fisetin)—physical mixture;
▪ SC 3 (20% fisetin)—sample treated with scCO_2_;
▪ PM 3 (20% fisetin)—physical mixture;
▪ SC 4 (25% fisetin)—sample treated with scCO_2_;
▪ PM 4 (25% fisetin)—physical mixture.

### 2.2. Materials

The substances used in the preparation of the systems, including fisetin (FIS) (>95%) and Poly(1-vinylpyrrolidone-co-vinyl acetate (CPV)), were obtained from Sigma Aldrich Chemie (Berlin, Germany).

Trifluoric acid and methanol HPLC grades were provided by Merck (Darmstadt, Germany). High-quality pure water was prepared using a Direct-Q 3 UV purification system (Millipore, Molsheim, France). 

Activity testing reagents: 2,2-Diphenyl-1-picrylhydrazyl, iron (III) chloride hexahydrate, 2,2′-casino-bis (3-ethylbenzothiazoline-6-sulfonic acid), neocuproine, 2,4,6-Tri(2-pyridyl)-s-triazine, and Trolox were supplied by Sigma-Aldrich (Schnelldorf, Germany). Sodium chloride and sodium hydrogen phosphate were purchased from Avantor Performance Materials (Gliwice, Poland). Ammonium acetate (NH_4_Ac) and methanol were supplied by Chempur (Piekary Śląskie, Poland). Cupric chloride dihydrate, ethanol (96%), isopropanol (99%), acetic acid (99.5%), and sodium acetate trihydrate were obtained from POCH (Gliwice, Poland).

### 2.3. Identification and Stability of Fisetin Delivery System 

PXRD analysis was performed at ambient temperature using a Bruker D2 Phaser (Bruker, Billerica, MA, USA) diffractometer with a LynxEye XE-T 1-dim detector (Bruker, Billerica, MA, USA) and Cu Kα radiation (λ = 1.54056 Å, generator setting: 40 kV and 40 mA). Diffraction data were collected at the 2θ scanning range between 5° and 40° with a step size of 0.02° and a counting time of 2 s/step.

The FT-IR spectra were obtained using an IRTracer-100 spectrophotometer (Shimadzu Corp., Kyoto, Japan). All spectra were measured between 400 and 4000 cm^−1^ in absorbance mode. The following spectrometer parameters were used: resolution: 4 cm^−1^, number of scans: 400, apodisation: Happ–Genzel. Each sample was placed directly on the ATR crystal. The solid samples were pressed against the ATR crystal, and the FT-IR spectrum was measured.

The stability analysis conducted for the FIS delivery systems was subjected to conditions simulating real-world storage. The samples were stored under controlled environmental conditions: 25 °C temperature and 70% relative humidity (RH), reflective of typical storage conditions. The assessment spanned three months to emulate an extended storage period.

To ensure the reliability of the stability study, the samples were meticulously preserved in sealed Falcon tubes, mitigating potential external influences, and maintaining a controlled environment. Moreover, the storage containers were strategically placed away from direct light exposure, minimising the impact of light-induced degradation.

### 2.4. Pharmaceutical Properties of Fisetin Delivery Systems

The test was performed to characterise solubility for the obtained systems. First, 10 mL of water was added to a 20 mL flat-bottom conical flask. Then, one of the obtained systems was added to each flask, and pure FIS was added to the last flask as a control. The flasks prepared this way were tightly closed to reduce water evaporation and placed in an incubator at 25 °C for 24 h with continuous stirring at 200 rpm. After incubation, 2.0 mL of the solution was withdrawn from each flask and filtered into the vial using a nylon syringe filter with a pore diameter of 0.22 µm. The obtained samples were analysed using HPLC with the previously described method.

All determinations were performed in three independent tests; three independent samples were taken from each, so each system was subjected to nine HPLC analyses.

The change in dissolution rate of the obtained FIS delivery systems was examined by determining the dissolution rate profiles, following the requirements of the European Pharmacopoeia at 37 ± 0.5 °C, using a paddle apparatus (Agilent, Santa Clara, CA, USA) with a paddle rotation speed of 50 rpm. As the acceptor dissolution media, 0.1 mol/L hydrochloric acid (pH ~ 1.2) and phosphate buffer (pH ~ 6.8) were used in the pH range corresponding to the gastrointestinal tract environment in 500.0 mL. FIS and the obtained systems were weighed into gelatin capsules and then placed in a sinker to prevent capsule flotation on the liquid’s surface. Dissolution samples were taken at appropriate time points. The samples were filtered through 0.22 μm nylon membrane syringe filters.

Dissolution profiles were compared using the model proposed by Moore and Flanner, previously described [37,38]. The evaluation of changes in the permeation of substances was carried out using the parallel test of permeability through artificial membranes (PAMPA, Parallel Artificial Membrane Permeability Assay) of gastrointestinal permeation. The received values of apparent permeability were compared using ANOVA variance analysis. The parallel permeability test through artificial biological membranes (PAMPA) served as an in vitro model of passive intercellular penetration. For the study of permeability study, saturated solutions were obtained after the previously described solubility method. The prepared solutions were filtered through 0.2 μm pore size filters. 

The PAMPA test consists of 96-well filter microplates divided into donor and acceptor chambers, separated by a 120 μm microfilter, and coated with a 20% lecithin solution. The plates were incubated at 37 °C for 60 min minutes. After incubation in the donor and acceptor parts, the carvedilol phosphate concentration was determined using the UHPLC-DAD method. The assessment of the effect of a substance on penetration was determined by comparing the values of apparent permeability coefficients using the following equation:

$$P_{app}=\frac{-\ln\left(1-\frac{C_{A}}{C_{equilibrium}}\right)}{S\times \left(\frac{1}{V_{D}}+\frac{1}{V_{A}}\right)\times t}$$

where *V_D_*—donor volume, *V_A_*—acceptor volume, $C_{equilibrium}=\frac{C_{D}\times V_{D}+C_{A}\times V_{A}}{V_{D}+V_{A}}$, *S*–membrane area, *t*—incubation time (in seconds).

Compounds with a *P_app_* < 1 × 10^−6^ cm/s are classified as having low permeability, and compounds with a *P_app_* > 1 × 10^−6^ cm/s are classified as having high permeability [39].

### 2.5. Biological Properties of the Fisetin Delivery System

In order to evaluate antioxidant activity, the ABTS, CUPRAC, DPPH, and FRAP inhibition assays were determined according to the previously reported methods [38,40,41].

The ABTS assay:

Green cation radicals were generated from ABTS by potassium persulfate. Subsequently, 10.0 μL of FIS and system solutions and 200.0 μL of ABTS•+ solution were added to 96-well plates. After 10 min of incubation at room temperature with shaking, absorbance was measured at λ = 734 nm. The ABTS scavenging activity was calculated using the formula: 

ABTS scavenging activity (%) = (A0 − A1)/A0 × 100%.

Trolox was used as a standard (Figure S1).

The CUPRAC assay:

Neocuproine and copper ion (II) complex interacted with the FIS solution to assess its antioxidant potential. A CUPRAC reagent was prepared by mixing acetate buffer, neocuproine, and CuC_l2_·H_2_O. Subsequently, 50.0 µL of the system solutions and 150.0 µL of the CUPRAC reagent were applied to a 96-well plate and incubated for 30 min at room temperature. Absorbance was measured at 450 nm, and the results were expressed as Trolox equivalents (Figure S2).

The DPPH assay:

Aqueous solutions were mixed with the DPPH solution, and the reaction mixture was incubated in the dark. Absorbance was measured at 517 nm. DPPH scavenging activity was calculated using the formula: 

DPPH scavenging activity (%) = (A0 − A1)/A0 × 100%. 

The results were expressed as Trolox equivalents (Figure S3).

The FRAP assay:

The FRAP assay involved mixing tested extracts with FRAP reagent and incubating the mixture at 37 °C. Absorbance was read at 593 nm, and the results were expressed as Trolox equivalents (Figure S4). 

While the neuroprotective assays were based on AChE and BuChE inhibition, they were determined according to the previously reported method [42]. The activity was then converted to galanthamine equivalent using the standard curves for AChE and BChE (Figures S5 and S6).

The assessment of microbiological activity was determined as follows: bacteria and yeast suspensions, protected and stored at −20 °C, were thawed at room temperature and then transferred to test tubes containing 10 mL of broth medium with 2% glucose (Oxoid). The cultures were carried out at 35 °C ± 2 °C for 24 h. In order to obtain a transparent turf layer, Mueller-Hilton Agar (Graso) solidified on Petri dishes was inoculated with a suspension of strains with a density of 10^6^ cfu/ml. The inoculated media were left for 15 min to absorb the microorganisms on their surface. The list of microorganisms used in the microbiological tests is given in Table 1.

The spot and well methods were employed to determine the activity of the samples. In the case of the spot method, 10 µL samples were applied to the surfaces of plates inoculated with standardised suspensions of bacteria and yeast (the spot method). In the case of the well method, wells with a diameter of 10 mm were cut in the inoculated media, and 100.0 µL of test samples were introduced into them. The plates with the samples were incubated at 35 °C ± 2 °C for 24 h. After incubation, the antimicrobial activity was assessed—based on measurements/observations of clear zones (inhibition) formed around the applied samples (the point method) or wells (the well method). The diameters of the brightening (inhibition) zones were measured using the Computer Scanning System (MultiScaneBase v14.02). The results were expressed in millimetres.

### 2.6. Statistical Analysis 

Statistical analysis was performed using Statistica 13.3 software (TIBCO Software Inc., Palo Alto, CA, USA). The Shapiro–Wilk test was implemented to check data distribution normality. Statistical significance was performed using a one-way analysis of variance (ANOVA) followed by Tukey’s HSD test. Measurements were considered significant when comparing FIS and the delivery systems at *p* < 0.05.

## 3. Results and Discussion

Preparing FIS delivery systems, a pivotal facet of this study, involved using supercritical carbon dioxide (scCO_2_) as a solvent. This method was meticulously designed to address the challenges associated with FIS’s poor solubility, aiming to unlock its therapeutic potential more effectively. In a controlled environment, FIS and carefully selected functional carriers were co-dissolved in scCO_2_, marking the inception of a journey toward the creation of advanced delivery systems. The choice of scCO_2_ as a solvent was strategic, aligning with the growing emphasis on green and sustainable methodologies in pharmaceutical research. This supercritical fluid acts as a benign medium, circumventing the need for traditional, often less eco-friendly solvents. The controlled conditions during the dispersion process played a pivotal role, influencing the final structure and properties of the obtained systems.

The resulting FIS delivery systems exhibited distinct organoleptic characteristics. Visual examination revealed a fine, homogeneous powder with improved flow properties. The amorphous nature of the FIS delivery systems was confirmed by powder X-ray diffraction (PXRD) and Fourier-transform infrared spectroscopy (FT-IR) analysis, highlighting the change in the crystalline structure under the influence of scCO_2_.

**Figure 2:**
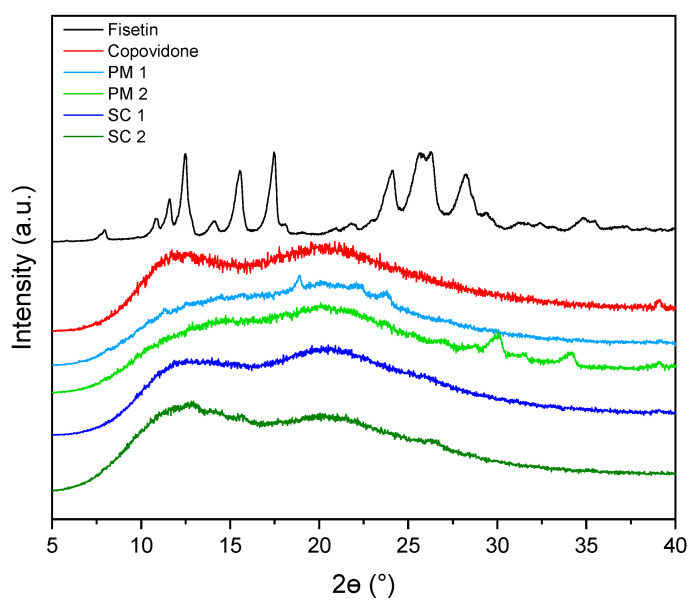
Diffractograms showing obtained amorphous dispersion of FIS.

PXRD patterns provide valuable insights into the FIS and copovidone crystal structure, the obtained physical mixtures (PM), and the systems treated with scCO_2_ (SC). In the PXRD pattern of crystalline form, well-defined peaks can be observed at specific 2θ angles, corresponding to the diffraction angles of the X-ray beam interacting with the crystal lattice (Figure 2). 

These peaks represent constructive interference of X-rays reflected from the crystal planes. The most prominent peak is typically observed around 5.7°–6.0° (2θ), corresponding to the (100) crystal plane, while other significant peaks appear at approximately 9.5°–10.0° (2θ), 14.6°–15.2° (2θ), 17.3°–18.0° (2θ), and 23.0°–23.7° (2θ), corresponding to other crystal planes such as (010), (110), (200), and (020), respectively [43,44]. These peaks provide information about the arrangement and orientation of atoms within the FIS crystal lattice. The presence of sharp and well-defined peaks confirms the crystalline nature of FIS. In contrast, amorphous CPV lacks distinct diffraction peaks in its PXRD pattern [45,46]. Instead, a broad hump or halo is observed, indicating the absence of a well-defined crystalline lattice (Figure 2). The PXRD pattern reflects the disordered arrangement of polymer chains within amorphous CPV, with no sharp peaks corresponding to specific crystal planes. The absence of sharp peaks suggests that the polymer chains lack long-range structural order and are randomly oriented [47].

When FIS is mixed physically with CPV, the PXRD pattern of the mixture exhibits the characteristic diffraction peaks of both components. However, the intensity and sharpness of the FIS peaks are reduced compared to pure FIS, indicating a decrease in crystallinity. CPV disrupts FIS’s crystalline structure, leading to a less ordered arrangement of FIS molecules within the mixture. This reduction in crystallinity can influence the physicochemical properties of the mixture, potentially affecting dissolution behaviour, solubility, and stability [32,48]. It may also contribute to improved bioavailability and therapeutic efficacy. Two systems were investigated regarding the amorphization of FIS using scCO_2_. Complete amorphization was achieved in the SC 1 system with 10% FIS, as confirmed by the absence of distinct diffraction peaks in the PXRD pattern. This indicates the crystalline lattice’s disruption and FIS’s conversion into an amorphous form [49,50]. In the SC 2 system with 15% FIS, partial amorphization was observed, with residual crystalline components present, as evidenced by diffraction peaks in the PXRD pattern. The higher FIS concentration in the SC 2 system hindered the complete disruption of the crystalline structure, resulting in a less pronounced amorphous form. Further investigations were not pursued in the SC 3 and SC 4 systems, as amorphization was uncertain, even with higher FIS concentrations of 20% and 25%, respectively.

**Figure 3:**
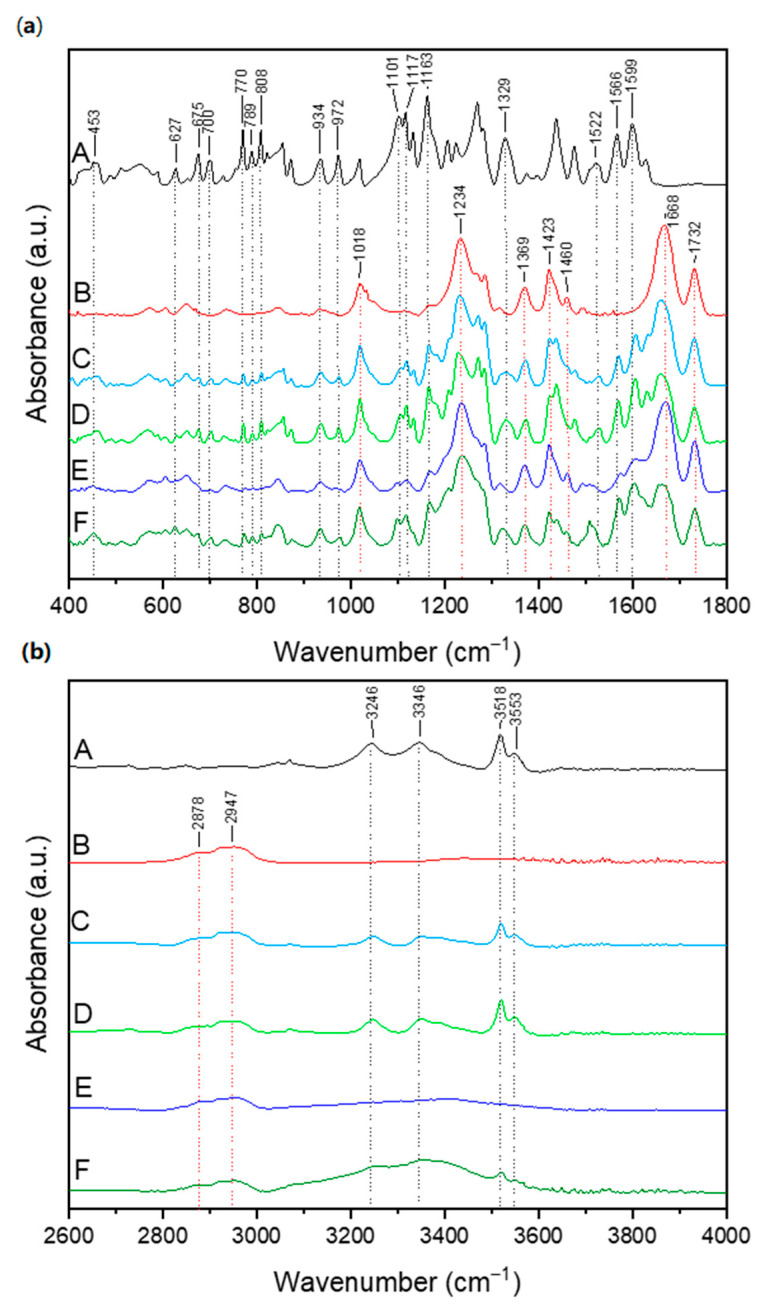
FT-IR analysis: (**a**) range 400–1800 cm^−1^, (**b**) range 2600–4000 cm^−1^. Legend: A—fisetin, B—copovidone, C—PM 1, D—PM 2, E—SC 1, F—SC 2. Dotted-line corresponds to the wavelength of the observed band, the numerical value is the exact value of the wavelength at which the highest intensity of the peak was observed.

The Fourier-transform infrared spectroscopy (FT-IR) results, as shown in Figure 3, provide valuable information about the molecular structure and functional groups present in the studied compounds. FT-IR spectroscopy is a powerful analytical technique that measures the absorption of infrared radiation by the sample, revealing characteristic vibrational modes of chemical bonds [51]. The observed peaks and bands in the presented FT-IR spectra represent the molecules’ specific vibrations in FIS, CPV, and their physical mixtures. By analysing these spectral features, we can gain insights into the molecular interactions, changes in chemical structure, and potential formation of new bonds or complexes. The FT-IR results will help us assess the compatibility between FIS and CPV, identify any shifts or alterations in the vibrational modes of the compounds upon mixing, and understand the effects of the physical mixtures on the functional groups present in the individual components.

The characteristic absorption bands of FIS raw powder appear at 808 cm^−1^ (out-of-plane C–H bending), 934 cm^−1^ (C-C-C bending), 972 cm^−1^ (C-C-C-H torsion), 1101 cm^−1^ (C-O stretching), 1117 cm^−1^ (C-OH stretching), 1163 cm^−1^ (C-OH stretching), 1329 cm^−1^ (C-OH deformation), 1522 cm^−1^ (C-C stretching), 1566 cm^−1^ (C-C stretching), 1599 cm^−1^ (aromatic ring vibration), 3246 cm^−1^ and 3346 cm^−1^ (OH stretching of the hydroxyl groups), 3518 cm^−1^ and 3553 cm^−1^ (stretching vibrations of phenolic O-H bonds) [52,53,54,55].

The characteristic absorption bands of CPV appear at 1020 cm^−1^ (C-N vibration), 1233 cm^−1^ (lactone structure), 1287 cm^−1^ (C-N stretching), 1369 cm^−1^ (CH_2_ bending), 1422 cm^−1^ (C-H vibration) 1458 cm^−1^ (O-H bending), 1665 cm^−1^ (C=O stretching of N-vinyl pyrrolidone), 1732 cm^−1^ C=O stretching of vinyl acetate, 2878 cm^−1^, and 2947 cm^−1^ (O-H stretching) [56,57]. 

The spectra recorded for physical mixtures combine bands from both FIS and CPV, confirming their lack of interactions. In contrast, the spectra of the systems obtained by the SFE method show differences like the spectrum, which indicate interactions between FIS and CPV. In both systems, we observe the disappearance of many characteristic FIS bands.

In both cases, bands characteristic of CPV predominate, suggesting that FIS has been dispersed in the polymer matrix. With the SC 1 system, we see much fewer characteristic FIS bands than with the SC 2 system. Many bands also have higher intensity; for example, 770, 789, 808, 934, 972, 1101, 1117, 1329, 1522, 1566, 1599, and 3518 cm^−1^. This suggests FIS is more dispersed in the SC 1 system than in the 10% system CPV matrix. This is consistent with the PXRD analysis, which indicates a fully amorphous system with 10% FIS content and an incomplete amorphous system with 15% FIS content. 

The decrease in the intensity of the bands corresponding to the C–O stretching (1101 cm^−1^), C-OH stretching (1117 cm^−1^, 1163 cm^−1^, and 1329 cm^−1^), and the disappearance of the OH stretching (3246 cm^−1^, 3346 cm^−1^, 3518 cm^−1^ and 3553 cm^−1^) bonds suggests that the C-O, C-OH and/or -OH groups of FIS may form hydrogen bonds with the C=O and/or -OH group of CPV, which are hydrogen bond acceptors.

The research on the pharmaceutical properties of the FIS delivery system included the analysis of the physical stability of amorphous dispersion, solubility in water, dissolution rate at pH 1.2 and 6.8, and penetration through the model cell membrane system (PAMPA) corresponding to absorption through the gastric, intestinal, and blood-brain barrier membranes.

**Figure 4:**
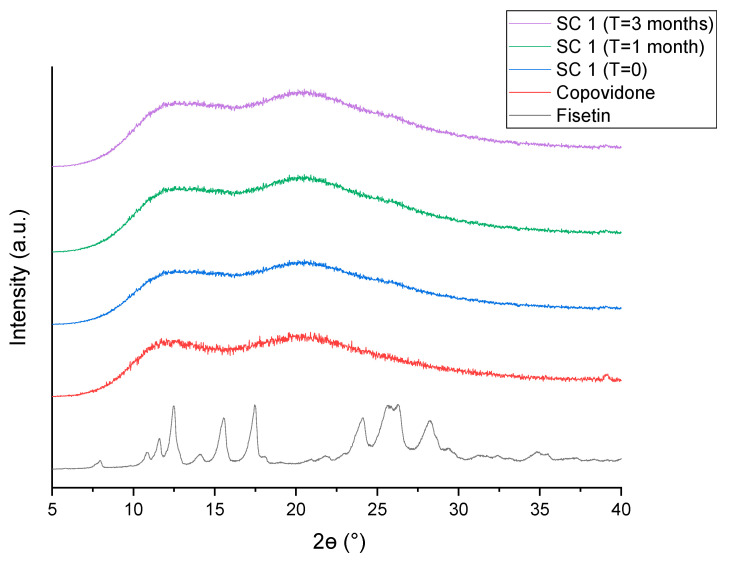
Diffractograms showing obtained amorphous dispersion of fisetin in delivery systems during stability studies.

The primary challenge in developing amorphous formulations lies in their inherent instability, stemming from the proclivity to transition from an energetically unfavourable amorphous state to a more stable crystalline configuration. To address this issue, a comprehensive analysis of the stability of the attained amorphous system was diligently conducted, scrutinising its behaviour during storage under ambient shelf conditions. The findings of this analysis revealed a remarkable outcome—the amorphous system remained stable and entirely amorphous throughout the storage period (Figure 4). Remarkably, this stability was achieved without employing additional protective measures for the formulated system.

In order to further substantiate the sustained integrity of the FIS delivery system, a meticulous solubility analysis was undertaken. This investigation aimed to verify the persistent high solubility of FIS (presumably a vital component of the formulation). The outcomes of this inquiry yielded affirmative results, confirming that the enhanced solubility observed in the initial formulation was impeccably preserved in the finalised amorphous system. In essence, these discerning analyses collectively underscore the success of the developed amorphous formulation, portraying it as a stable and reliably amorphous system even under the rigours of storage conditions. The corroborative solubility study serves to affirm not only the stability but also the functional efficacy of the formulation, particularly in sustaining the heightened solubility of FIS. This confluence of stability and enhanced solubility positions the developed amorphous system as a promising candidate for applications demanding both stability and increased solubility, marking a significant stride in advancing pharmaceutical formulations.

As presented in Table 2, the solubility results provide crucial information about FIS’s dissolution behaviour and its physical mixtures with CPV and SC systems. Solubility is a critical parameter directly impacting a drug’s or active compound’s bioavailability and therapeutic efficacy [58]. The solubility data in Table 2 reflects the amount of FIS and its physical mixtures dissolved in water under given conditions. By comparing the solubility values of FIS alone, with the presence of CPV, and under the influence of sCO_2_, the impact on the solubility properties of FIS can be evaluated.

The solubility of pure FIS is determined to be 1.00 × 10^−3^ ± 3.00 × 10^−4^ mg/mL. When FIS is subjected to scCO_2_ conditions, significant improvements in solubility are observed. In the SC 1 system, the solubility of FIS increases to 4.545 × 10^−1^ ± 5.00 × 10^−3^ mg/mL, representing a remarkable solubility improvement of 454.512%. Similarly, the SC 2 system shows a solubility enhancement to 3.415 × 10^−1^ ± 6.00 × 10^−4^ mg/mL, corresponding to a solubility improvement of 341.491%. In the PM of FIS with CPV, the solubility values also increase compared to pure FIS. The PM 1 system exhibits a solubility of 1.390 × 10^−1^ ± 1.30 × 10^−3^ mg/mL, representing an improvement of 138.9535%. The PM 2 system shows a 1.374 × 10^−1^ ± 1.20 × 10^−3^ mg/mL solubility, representing an improvement of 137.368%. The solubility improvements are slightly lower for the higher FIS concentrations investigated in the SC 3 and SC 4 systems. In the SC 3 system, the solubility of FIS is measured at 2.712 × 10^−1^ ± 5.00 × 10^−4^ mg/mL, corresponding to a solubility improvement of 271.224%.

The SC 4 system demonstrates a solubility of 2.361 × 10^−1^ ± 1.40 × 10^−3^ mg/mL; the utilisation of scCO_2_ resulted in a notable solubility enhancement by 236.056%. When comparing the improvements in solubility observed between the particulate microprecipitation (PM) and co-precipitation with CPV (Controlled Precipitation via Vaporisation), it is evident that the enhancements achieved in the supercritical scCO_2_ systems tend to be lower. This implies that the scCO_2_ method may be more efficacious in augmenting the solubility of FIS compared to the physical mixing approach with CPV. The observed enhancements in FIS solubility align with the findings in existing literature, underscoring the potential of scCO_2_ to enhance the solubility of otherwise poorly soluble compounds. The solubility enhancements achieved in this study support using scCO_2_ as a promising approach for improving FIS’s solubility and dissolution properties, which can ultimately enhance its bioavailability and therapeutic effectiveness. Additionally, the solubility improvements observed in the physical mixtures with CPV are consistent with the literature that highlights the ability of CPV to enhance the dissolution rates and solubility of various active pharmaceutical ingredients [59,60]. The presence of CPV in the physical mixtures contributes to the improved solubility of FIS, potentially by forming molecular dispersions or facilitating the disruption of FIS aggregates.

**Figure 5:**
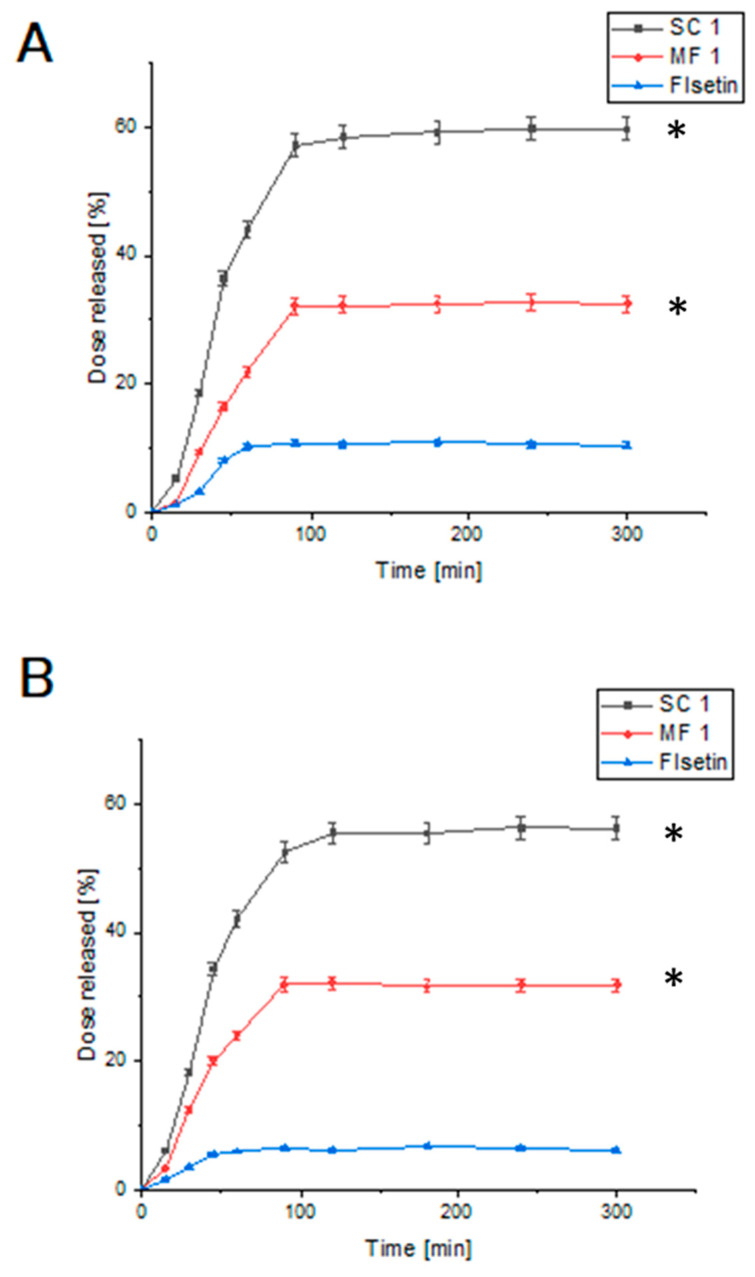
The dissolution rate of FIS and delivery systems obtained using scCO_2_ at pH 1.2 (**A**) and 6.8 (**B**). Data expressed as mean ± SD; *—significance at *p* ≤ 0.05.

The dissolution profiles of FIS in a solution with pH 6.8, representing the intestinal environment, were analysed and compared to the previous results obtained at pH 1.2, corresponding to the stomach environment (Figure 5).

The dissolution profiles of FIS in a solution with pH 6.8 (Figure 5B), representing the intestinal environment, were compared to the previous results obtained at pH 1.2 (Figure 5A), corresponding to the stomach environment. At the initial time of 15 min, the SC 1 system exhibited a dissolution rate of 6.244% ± 0.187% at pH 6.8, while PM1 showed a dissolution rate of 3.488% ± 0.11%. FIS alone had a dissolution rate of 1.764% ± 0.0661%. Comparing these values to the dissolution rates at pH 1.2, it is evident that they were generally lower in the intestinal environment. As the dissolution process progressed, the dissolution rates increased for all formulations at pH 6.8. At 30 min, the SC 1 system showed a dissolution rate of 18.277% ± 0.548%, PM 1 had a dissolution rate of 12.495% ± 0.395%, and FIS alone had a dissolution rate of 3.477% ± 0.13%. These dissolution rates were also lower compared to the corresponding time points at pH 1.2. Similar trends were observed at subsequent time points of 45, 60, 90, 120, 180, 240, and 300 min, where the dissolution rates at pH 6.8 were consistently lower than those at pH 1.2. The lower dissolution rates in the intestinal environment can be attributed to several factors. The pH difference between the stomach and intestine affects FIS’s solubility and dissolution behaviour [61]. FIS may have lower solubility in the relatively neutral pH of the intestine, leading to slower dissolution rates. Different enzymes and transporters in the gastrointestinal tract can also influence the dissolution and absorption of FIS. The comparison between the dissolution profiles at pH 1.2 (representing the stomach) and pH 6.8 (representing the intestine) highlights the importance of considering the physiological conditions of different gastrointestinal tract regions when formulating FIS for optimal dissolution and absorption.

**Figure 6:**
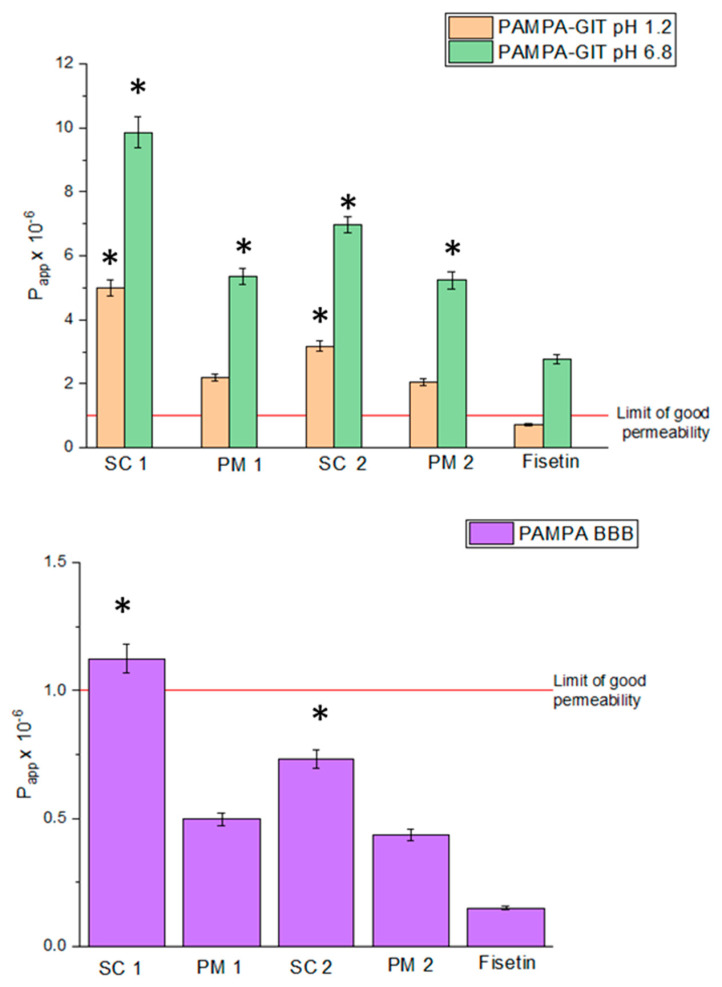
Permeability of FIS and delivery systems obtained using scCO_2_. Data expressed as mean ± SD; *—significance at *p* ≤ 0.05.

The results presented in Figure 6 provide insights into the permeability of FIS and its formulations using the PAMPA (Parallel Artificial Membrane Permeability Assay) model, which simulates different in vitro environments: gastric (pH 1.2), intestinal (pH 6.8), and the blood-brain barrier (BBB).

In the gastric environment (pH 1.2), the SC 1 system exhibited a permeability value of 5.01 × 10^−6^ ± 2.50 × 10^−7^ cm/s, indicating relatively good permeability. Similarly, PM 1 demonstrated permeability of 2.20 × 10^−6^ ± 1.10 × 10^−7^ cm/s. These results suggest that the solubility enhancements achieved through the SC 1 system and PM 1 contribute to improved permeability in the gastric environment compared to FIS alone (7.31 × 10^−7^ ± 3.65 × 10^−8^ cm/s). In the intestinal environment (pH 6.8), the SC 1 system showed a permeability value of 9.86 × 10^−6^ ± 4.93 × 10^−7^ cm/s, while PM 1 displayed a permeability of 5.36 × 10^−6^ ± 2.68 × 10^−7^ cm/s. Again, these values indicate enhanced permeability compared to FIS alone (2.77 × 10^−6^ ± 1.39 × 10^−7^ cm/s). Regarding the blood-brain barrier (BBB), the SC 1 system exhibited a permeability value of 1.12 × 10^−6^ ± 5.62 × 10^−8^ cm/s, while PM 1 demonstrated a 4.97 × 10^−7^ ± 2.48 × 10^−8^ cm/s permeability. These permeability values are relatively lower compared to the gastric and intestinal environments. Nevertheless, they still suggest improved permeability compared to FIS alone (1.50 × 10^−7^ ± 7.49 × 10^−9^ cm/s).

Overall, the results indicate that the solubility enhancements achieved through the SC 1 system and PM 1 contribute to improved permeability of FIS in the PAMPA in vitro model across different environments. The increased solubility likely enhances the availability and interaction of FIS with the artificial membrane, facilitating its permeation [62,63]. These findings suggest that the SC 1 system and PM 1 formulations can potentially enhance FIS’s bioavailability and pharmacokinetic profile. It is important to note that the PAMPA model provides a simplified representation of permeability and does not capture the complexities of in vivo absorption and distribution [64]. Further studies, including in vivo evaluations, are necessary to validate the observed permeability enhancements and assess the translational potential of these formulations.

**Figure 7:**
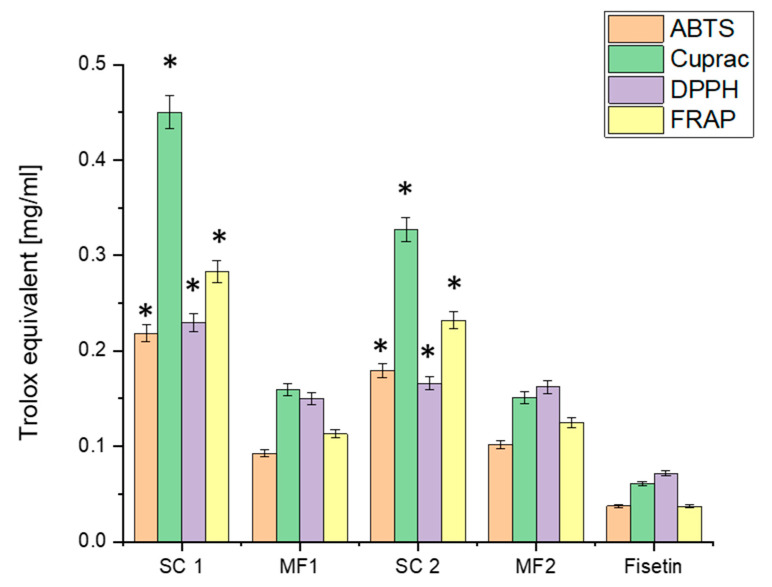
Antioxidant activity of FIS and delivery systems obtained using scCO_2_. Data expressed as mean ± SD; *—significance at *p* ≤ 0.05.

After assessing the solubility and permeability improvements achieved with FIS and its physical mixtures and systems, it becomes crucial to investigate the impact of these formulations on the antioxidant activity of FIS. Figure 7 presents the results of the antioxidant activity in four in vitro models. It is important to note that, following the solubility evaluation, understanding the potential enhancement in FIS’s antioxidant and biological activity is of significant interest. We anticipate enhancing its antioxidant activity by improving FIS’s solubility using techniques such as scCO_2_ or physical mixing with excipients like CPV. The solubility improvement may lead to increased bioavailability, improved cellular uptake, and enhanced antioxidant efficacy [38,65].

In the ABTS assay, the SC 1 system exhibited a significantly higher antioxidant activity (0.21832 ± 0.00873 mg/mL Trolox equivalent) than FIS alone (0.03744 ± 0.0015 mg/mL Trolox equivalent). This substantial increase indicates that the solubility enhancement achieved in the SC 1 system significantly improved the ability of FIS to scavenge ABTS radicals. Similarly, MF1 showed enhanced antioxidant activity (0.09235 ± 0.00369 mg/mL Trolox equivalent) compared to FIS alone. In the CUPRAC assay, the SC 1 system and PM 1 demonstrated improved antioxidant activity compared to FIS alone. The SC 1 system exhibited an antioxidant activity of 0.45023 ± 0.01747 mg/mL Trolox equivalent, while PM 1 showed an activity of 0.15944 ± 0.00619 mg/mL Trolox equivalent. These results further emphasise the solubility enhancement’s positive impact on FIS’s antioxidant capacity. The DPPH assay results also confirmed the enhanced antioxidant activity of FIS in the formulated systems. The SC 1 system exhibited an antioxidant activity of 0.22946 ± 0.00952 mg/mL Trolox equivalent, while PM 1 showed an activity of 0.14963 ± 0.00621 mg/mL Trolox equivalent. These findings suggest that the improved solubility of FIS in these systems enhances its ability to scavenge DPPH radicals effectively. In the FRAP assay, the SC 1 system and PM 1 demonstrated higher antioxidant activity than FIS alone. The SC 1 system exhibited an antioxidant activity of 0.28341 ± 0.01148 mg/mL Trolox equivalent, while PM 1 showed an activity of 0.11314 ± 0.00458 mg/mL Trolox equivalent. These results indicate that the solubility enhancements achieved through the formulated systems contribute to improved ferric-reducing antioxidant power.

Overall, the results consistently indicate that the solubility enhancements achieved through the SC 1 system and PM 1 significantly enhance the antioxidant activity of FIS in all four in vitro models. The improvements in antioxidant capacity observed in the SC 1 system and PM 1 highlight the importance of solubility in maximising FIS’s antioxidant potential. The enhanced antioxidant activity observed in the formulated systems can be attributed to FIS’s improved solubility and bioavailability, facilitating its interaction with reactive oxygen species and free radicals. These findings align with previous studies demonstrating the positive correlation between solubility enhancements and the antioxidant effects of natural compounds [66]. The significant improvements in the antioxidant activity of FIS achieved through the SC 1 system have important implications for potential therapeutic applications. The ability of FIS to scavenge free radicals and counteract oxidative stress is crucial for combating various diseases associated with oxidative damage, including neurodegenerative disorders [67,68]. Notably, the antioxidant activity of FIS and its formulations may vary depending on the specific in vitro models used and the concentrations evaluated. Therefore, further studies, including in vivo investigations, are necessary to validate the observed antioxidant effects and evaluate the translational potential of these formulations.

**Figure 8:**
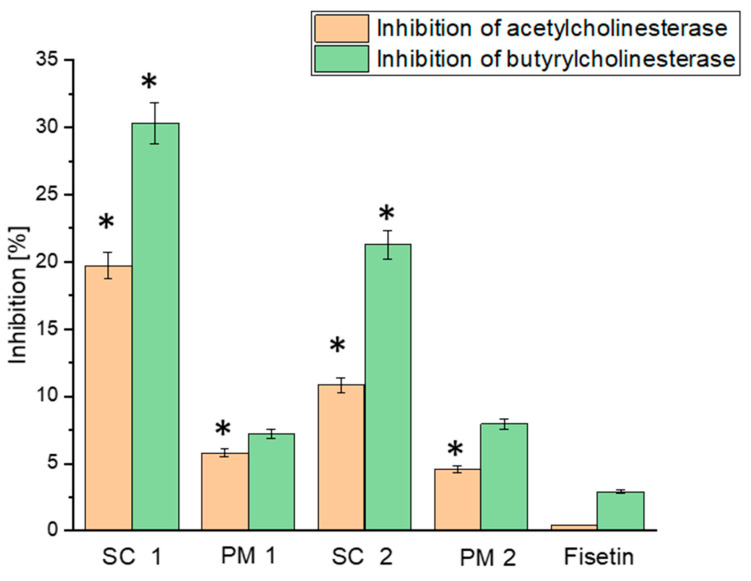
Neuroprotective activity of FIS and delivery systems obtained using scCO_2_. Data expressed as mean ± SD; *—significance at *p* ≤ 0.05.

The results presented in Figure 8 depict the neuroprotective activity of FIS and its formulations, as assessed through the inhibition of acetylcholinesterase (AChE) and butyrylcholinesterase (BuChE) enzymes. The neuroprotective activity is expressed as a percentage of enzyme inhibition, providing a basis for comparing and evaluating the compounds’ potential efficacy.

In the neuroprotection assays, the inhibitory effects of FIS and the formulated systems (the SC 1 system and PM 1) were evaluated against acetylcholinesterase (AChE) and butyrylcholinesterase (BuChE), enzymes involved in the breakdown of acetylcholine, a neurotransmitter critical for memory and cognitive functions [69,70]. FIS exhibited a low inhibitory effect on AChE and BuChE, with values of 0.42221% ± 0.02111% and 2.91558% ± 0.14578%, respectively. These results indicate that FIS possesses potent anti-cholinesterase activity, desirable for treating Alzheimer’s disease, with potential limitations resulting from low solubility. Inhibiting AChE and BuChE can increase acetylcholine levels in the brain, potentially improving cognitive function and memory [71]. When formulated in the SC 1 system, FIS showed enhanced neuroprotective effects compared to FIS alone. The inhibitory effects on AChE and BuChE for the SC 1 system were 2.4-fold and 6.9-fold higher, respectively, compared to FIS alone. This improvement in neuroprotective activity can be attributed to FIS’s enhanced solubility and bioavailability in the SC 1 system, allowing for better interaction with the target enzymes.

The neuroprotective effects of FIS and its formulated systems are of particular significance for Alzheimer’s disease, a neurodegenerative disorder characterised by the loss of cholinergic neurons and impaired cholinergic neurotransmission [72,73]. Inhibiting AChE and BuChE can help restore cholinergic function and potentially alleviate cognitive impairments associated with Alzheimer’s disease. The results support the potential therapeutic value of FIS as a natural compound with neuroprotective effects. The enhanced neuroprotective activity observed in the solubility-enhanced formulation (the SC 1 system) highlights the importance of formulating FIS to improve its bioavailability and therapeutic efficacy. Further studies are warranted to explore the mechanisms of action underlying the neuroprotective effects of FIS and its formulated systems. In addition, in vivo, studies and clinical trials are necessary to assess FIS’s efficacy, safety, and long-term effects for treating Alzheimer’s disease.

The results of the antimicrobial activity assays reveal the potential of FIS and its formulated systems (SC 1, PM 1, SC 2, and PM 2) against a panel of indicator strains (Table 3).

The results show that the SC 1 system exhibits bactericidal activity against *E. coli*, *P. aeruginosa*, and *C. albicans*. It also shows slight activity against *S. aureus* and *B. subtilis*. On the other hand, the SC 2 system demonstrates feeble activity against *C. albicans* and *P. aeruginosa*, which is almost invisible in the images. Additionally, the SC 2 system exhibits bacteriostatic effects on *P. aeruginosa*. However, the PM 2 and SC 2 systems show lower activity, as indicated by smaller inhibition zones, than the SC 1 system. The remaining samples do not demonstrate any antagonistic activity against the tested microorganisms. These findings provide insights into the potential antimicrobial properties of the tested formulations, particularly the SC 1 system, in the context of Alzheimer’s disease and its multifaceted impact on the microbiome. Alzheimer’s disease is characterised by neurodegeneration and cognitive decline, with emerging research suggesting a link between the gut microbiome and this neurodegenerative disorder [74,75].

The enhanced antimicrobial efficacy of SC 1 can be attributed to the substantially increased solubility associated with its amorphous form. This aligns with the principle that amorphous structures often exhibit improved solubility compared to their crystalline counterparts. The higher solubility of SC 1 leads to elevated concentrations of fisetin, facilitating a more pronounced antimicrobial effect. Conversely, the physical mixture, PM 1, demonstrated no antimicrobial activity against the tested strains. This can be attributed to its components’ distinct physical and chemical properties, which may limit the release and availability of fisetin for interaction with the microbial strains. The findings on SC 1 underscore the importance of considering the structural aspects of drug delivery systems in pharmaceutical research. The amorphous nature of SC 1 plays a pivotal role in influencing its bioavailability and, consequently, the antimicrobial activity of fisetin.

Dysbiosis, an imbalance in the gut microbiota, has been observed in individuals with Alzheimer’s. Disruptions in the gut microbiome can produce harmful metabolites and pro-inflammatory molecules, triggering chronic systemic inflammation and potentially contributing to neuroinflammation and neurodegeneration [76,77]. Consequently, restoring a balanced and healthy gut microbiota may have therapeutic implications for Alzheimer’s disease. The antimicrobial activity observed in the SC 1 system, particularly against *E. coli*, *P. aeruginosa*, and *C. albicans*, is intriguing in the context of Alzheimer’s disease. An essential aspect of the obtained results is the lack of negative impact of the tested systems and FIS itself on health-promoting probiotic strains, which indicates targeted action only against potentially pathogenic bacterial strains. Dysbiosis and gut dysfunctions have been associated with an increased risk of Alzheimer’s disease and the exacerbation of cognitive impairments. Addressing dysbiosis and targeting specific pathogenic microorganisms may be essential in restoring gut homeostasis and potentially mitigating neuroinflammation and neurodegeneration [78]. Furthermore, the multifaceted effects of the tested formulations on both neuroprotection and the gut microbiota highlight their potential for comprehensive therapeutic interventions. The neuroprotective properties of FIS and its ability to modulate the gut microbiota, as demonstrated by the SC 1 system, present an intriguing opportunity to address the complex interactions between the gut-brain axis, neurodegeneration, and dysbiosis in Alzheimer’s disease.

## 4. Conclusions

This extensive investigation delves into the neuroprotective potential of fisetin within its amorphous delivery system, a breakthrough achieved through the innovative scCO_2_ amorphization process. The transformation of crystalline fisetin into its amorphous state has proven to be a game-changer, significantly enhancing its solubility and bioavailability. The outstanding attributes of amorphous fisetin, including superior dissolution characteristics, position it as a potentially impactful element in slowing disease progression and preserving cognitive function. One of the formidable challenges in developing amorphous formulations is their intrinsic instability, often reverting to a more stable crystalline state. Our study presents an exceptional outcome—the amorphous system remained consistently stable throughout the storage period, eliminating the need for additional protective measures. This stability is crucial in ensuring the feasibility of deploying amorphous fisetin in practical pharmaceutical applications. Crucially, fisetin exhibited notable neuroprotective properties, particularly relevant in addressing neurodegenerative disorders such as Alzheimer’s disease. The reduction in crystallinity observed in physical mixtures with a polymer excipient suggests fisetin’s potential for improved pharmaceutical availability, highlighting its therapeutic promise in neurodegenerative disease treatment. Our in-depth in vitro studies spotlighting fisetin’s antioxidant activity underscore its role in mitigating oxidative stress, a pivotal factor in neurodegenerative conditions.

Furthermore, our exploration of the gut-brain axis and microbiome modulation unveils fisetin’s potential for Alzheimer’s therapy. Fisetin can modulate the gut microbiome, particularly in its amorphous form, offering a strategic approach to restoring a healthy microbial community and alleviating inflammatory processes. The observed antimicrobial activity in formulations such as the SC 1 system suggests a potential avenue for addressing dysbiosis and neuroinflammatory implications. This multifaceted approach positions fisetin as a versatile candidate for tackling various aspects of neurodegenerative disorders beyond its direct neuroprotective effects. 

In conclusion, fisetin, especially in its amorphous state, emerges as a robust candidate for neuroprotection, boasting multifaceted attributes such as improved solubility, antioxidant prowess, and microbiome intervention. While these insights provide a solid foundation, we acknowledge that further research and clinical studies are imperative to translate these promising results into tangible clinical applications. This advancement brings us closer to effective interventions for neurodegenerative disorders, addressing a critical need in the field of neurology.

## Figures and Tables

**Table 1:** Microorganisms are used as indicators to assess the tested samples’ antimicrobial activity (killing and/or static).

Microorganism	Category	Origin
Gram-positive bacteria		
*Bacillus subtilis*	Spore-forming bacteria causing food spoilage	Isolate from food
*Enterococcus faecalis*	Antibiotic-resistant fecal bacteria; indicator of fecal contamination	Isolate from food
*Listeria monocytogenes*	Pathogenic bacteria; parasite of animals and humans	ATCC 19111
*Staphylococcus aureus*	Pathogenic bacteria with enterotoxigenic properties	ATCC 25923
*Lacticaseibacillus rhamnosus* (*Lactobacillus rhamnosus*) GG	Probiotic bacteria	ATCC 53103
*Lacticaseibacillus paracasei* (*Lactobacillus paracasei*)	Probiotic bacteria	CNCM I-1572
*Lactiplantibacillus plantarum* (*Lactobacillus plantarum*) 299v	Probiotic bacteria	Isolate from commercial product SanProbi IBS
Gram-negative bacteria		
*Escherichia coli*	Intestinal bacteria; sanitary indicator	ATCC 10536
*Pseudomonas aeruginosa*	Pathogenic bacteria resistant to antibiotics	ATCC 15442
*Salmonella enterica*	Pathogenic bacteria causing food poisoning	Clinical isolate from WSSE
Yeast		
*Candida albicans*	Pathogenic yeast	Isolate from faeces

**Table 2:** Solubility of FIS and delivery systems obtained using scCO_2_.

System	Solubility [mg/mL]	Solubility Improvement [%]
Fisetin	1.00 × 10^−3^ ± 3.00 × 10^−4^	X
SC 1 system	4.545 × 10^−1^ ± 5.00 × 10^−3^	454.512
PM 1	1.390 × 10^−1^ ± 1.30 × 10^−3^	138.953
SC 2 system	3.415 × 10^−1^ ± 6.00 × 10^−4^	341.491
PM 2	1.374 × 10^−1^ ± 1.20 × 10^−3^	137.368
SC 3 system	2.712 × 10^−1^ ± 5.00 × 10^−4^	271.224
PM 3	1.346 × 10^−1^ ± 1.10 × 10^−3^	134.632
SC 4 system	2.361 × 10^−1^ ± 1.40 × 10^−3^	236.056
PM 4	1.388 × 10^−1^ ± 9.00 × 10^−4^	138.811

**Table 3:** Antimicrobial activity of FIS and delivery systems obtained using scCO_2_.

Indicator Strains	Activity [mm]/Sample				
	SC 1 System	PM 1	SC 2 System	PM 2	Fisetin
*Bacillus subtilis*	+/2 *	0	0	0	0
*Enterococcus faecalis*	0	0	0	0	0
*Listeria monocytogenes*	0	0	0	0	0
*Staphylococcus aureus*	+/3 *	0	0	0	0
*Lacticaseibacillus rhamnosus* GG	0	0	0	0	0
*Lactiplantibacillus plantarum* 299v	0	0	0	0	0
*Lacticaseibacillus paracasei*	0	0	0	0	0
*Escherichia coli*	+/6 *	0	+/2 *	+/− *	0
*Pseudomonas aeruginosa*	+/4 *	0	0	0	0
*Salmonella enterica*	0	0	0	0	0
*Candida albicans*	+/5 *	0	+/2 *	0	0

* + means the demonstration of antibacterial activity along with the area of inhibition; +/− means the absence of values, only growth inhibition was demonstrated at the site of the sample application.

## Data Availability

The data are available in the manuscript and the supplementary materials.

## Supplementary Materials

The following supporting information can be downloaded at: https://www.mdpi.com/article/10.3390/antiox13010024/s1. Figure S1: standard curve for the conversion of antioxidant activity in the FRAP assay for Trolox; Figure S2: standard curve for the conversion of antioxidant activity in the CUPRAC assay for Trolox; Figure S3: standard curve for the conversion of antioxidant activity in the DPPH assay for Trolox; Figure S4: standard curve for the conversion of antioxidant activity in the ABTS assay for Trolox; Figure S5. Acetylcholinesterase Inhibition Standard Curve for Galantamine; Figure S6. Butyrylcholinesterase Inhibition Standard Curve for Galantamine.
