# Supplementary material for: A Fisetin Delivery System for Neuroprotection: A Co-Amorphous Dispersion Prepared in Supercritical Carbon Dioxide
Source: Antioxidants (Basel). 2023 Dec 21;13(1):24. doi: 10.3390/antiox13010024 (PMC10812833; doi:10.3390/antiox13010024)
Supplement: Supplementary file 1 [file antioxidants-13-00024-s001.zip › antioxidants-2782834-supplementary.pdf]

antioxidants

MDPI

Article

Supercritical Carbon Dioxide-Induced Amorphous Fisetin: A Promising Candidate for Alzheimer's Therapy and Microbiome Modulation

Szymon Sip [1], Natalia Rosiak [1], Anna Sip [2] and Judyta Cielecka-Piontek [1,*]

[1] Department of Pharmacognosy and Biomaterials, Poznan University of Medical Sciences, Rokietnicka 3, 60-806 Poznan, Poland; szymonsip@ump.edu.pl
[2] Department of Biotechnology and Food Microbiology, Poznan University of Life Sciences, Wojska Polskiego 48, 60-627 Poznan, Poland; anna.sip@up.poznan.pl
* Correspondence: jpiontek@ump.edu.pl

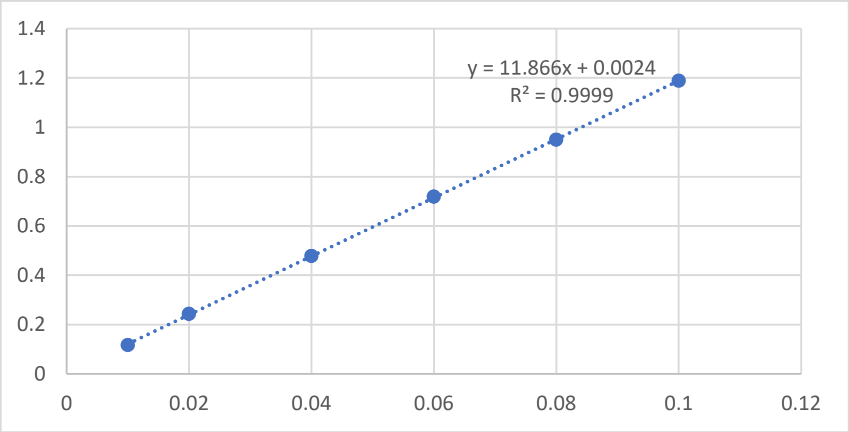

Figure S1. Standard curve for the conversion of antioxidant activity in the FRAP assay for Trolox

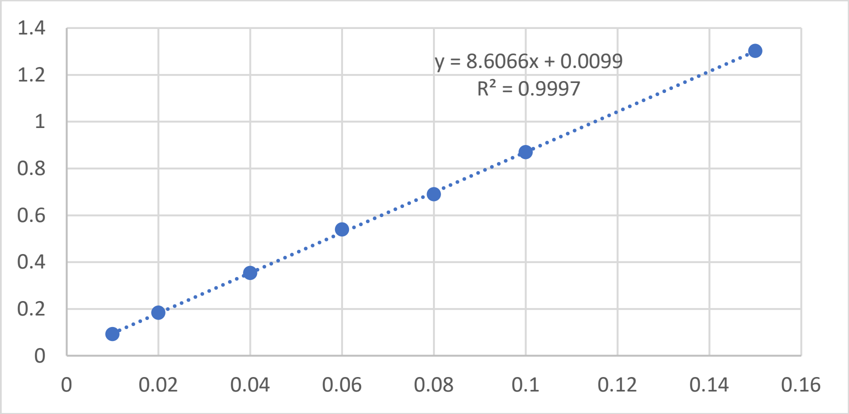

Figure S2. Standard curve for the conversion of antioxidant activity in the CUPRAC assay for Trolox

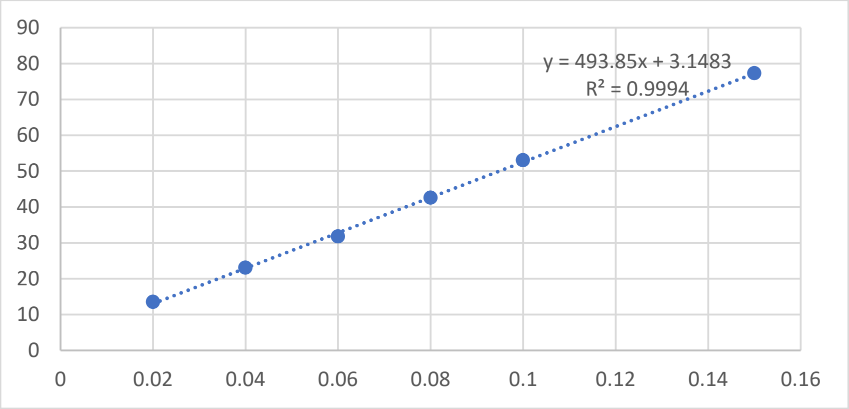

Figure S3. Standard curve for the conversion of antioxidant activity in the DPPH assay for Trolox

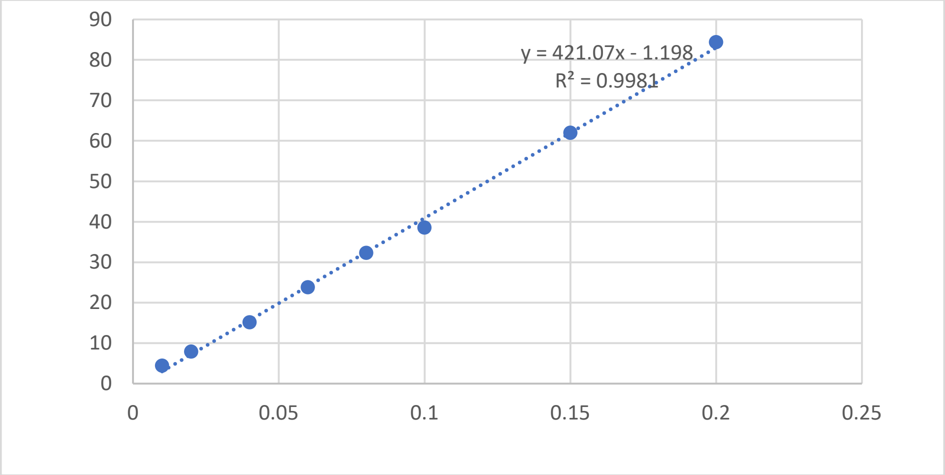

Figure S4. Standard curve for the conversion of antioxidant activity in the ABTS assay for Trolox

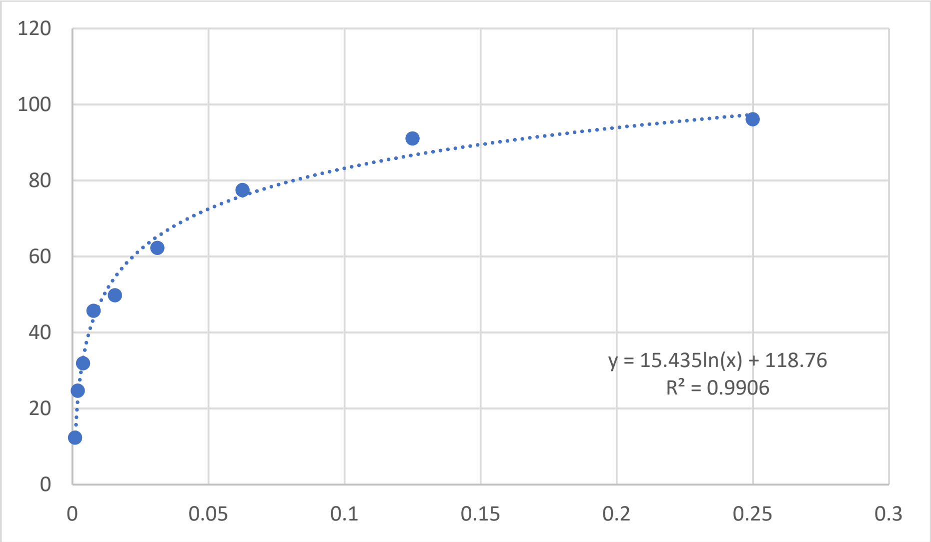

Figure S5. Acetylcholinesterase Inhibition Standard Curve for Galantamine

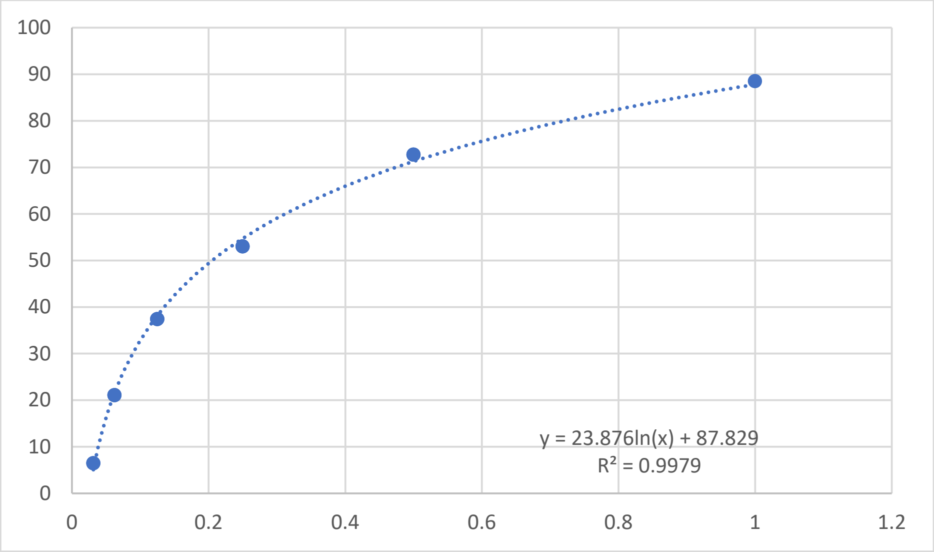

Figure S6. Butyrylcholinesterase Inhibition Standard Curve for Galantamine
